# Trends in national and county-level Hispanic mortality in the United States, 2011–2020

**DOI:** 10.1038/s41598-022-15916-x

**Published:** 2022-07-12

**Authors:** Ashish Sarraju, Summer Ngo, Melanie Ashland, David Scheinker, Fatima Rodriguez

**Affiliations:** 1 Division of Cardiovascular Medicine and Cardiovascular Institute, Stanford University School of Medicine, Stanford, CA USA; 2 Stanford Cancer Institute, Stanford University School of Medicine, Stanford, CA USA; 3 Clinical Excellence Research Center, Stanford University School of Medicine, Stanford, CA USA; 4 Division of Cardiovascular Medicine, Center for Academic Medicine, Stanford University, 453 Quarry Road, Stanford, CA 94304 USA

**Keywords:** Health care, Public health, Diseases

## Abstract

Hispanic populations generally experience more adverse socioeconomic conditions yet demonstrate lower mortality compared with Non-Hispanic White (NHW) populations in the US. This finding of a mortality advantage is well-described as the “Hispanic paradox.” The Coronavirus Disease 2019 (COVID-19) pandemic has disproportionately affected Hispanic populations. To quantify these effects, we evaluated US national and county-level trends in Hispanic versus NHW mortality from 2011 through 2020. We found that a previously steady Hispanic mortality advantage significantly decreased in 2020, potentially driven by COVID-19-attributable Hispanic mortality. Nearly 16% of US counties experienced a reversal of their pre-pandemic Hispanic mortality advantage such that their Hispanic mortality exceeded NHW mortality in 2020. An additional 50% experienced a decrease in a pre-pandemic Hispanic mortality advantage. Our work provides a quantitative understanding of the disproportionate burden of the pandemic on Hispanic health and the Hispanic paradox and provides a renewed impetus to tackle the factors driving these concerning disparities.

## Introduction

Although Hispanic populations in the United States (US) generally experience higher poverty, lower educational attainment, and a greater likelihood of being uninsured, they have lower mortality than Non-Hispanic White (NHW) populations—an epidemiological observation termed the “Hispanic paradox”^1,2^. In a systematic review and meta-analysis of 58 studies and more than 4 million individuals, Hispanic populations had a 17.5% lower risk of mortality as compared with non-Hispanic white populations^1^. This mortality advantage was observed in healthy groups as well as groups with pre-existing health conditions including cardiovascular disease.

Possible explanations of the Hispanic paradox include the protective role of culture, including residence in ethnic enclaves, social cohesion, and healthier dietary patterns; migration flow patterns relating to emigration of Hispanic individuals out of the US before death (also known as the salmon bias hypothesis) or the migration of predominantly healthier Hispanic individuals to the US (also known as the healthy migrant hypothesis); and methodological issues relating to reporting and misclassification errors in mortality statistics by race/ethnicity^2–4^. While it remains uncertain to what degree these various considerations can explain the observed mortality advantage, the presence of the Hispanic paradox has generally been described in studies across a range of health outcomes.

Tracking the Hispanic mortality advantage may provide key insights into ethnicity-specific population health trends. After the COVID-19 pandemic, an unprecedented county-level reversal of the Hispanic paradox was observed in Los Angeles (LA) County, where Hispanic mortality increased exceeded NHW mortality in 2020^5^. This mortality advantage reversal observed in LA county highlighted the magnitude of the previously described overrepresentation of Hispanic individuals in COVID-19 infections, hospitalizations, and deaths^6,7^. However, it is not known whether similar post-pandemic reversals of the Hispanic mortality advantage occurred at a national level. Quantifying post-pandemic changes in Hispanic mortality broadly across US counties is a crucial first step to identify and tackle the structural factors driving these disparities.

We therefore sought to evaluate trends in US national and county-level mortality in Hispanic and NHW individuals through 2020 to identify post-pandemic reversals of the Hispanic mortality advantage which may have key implications for national and county-level public health.

## Results

**Figure 1:**
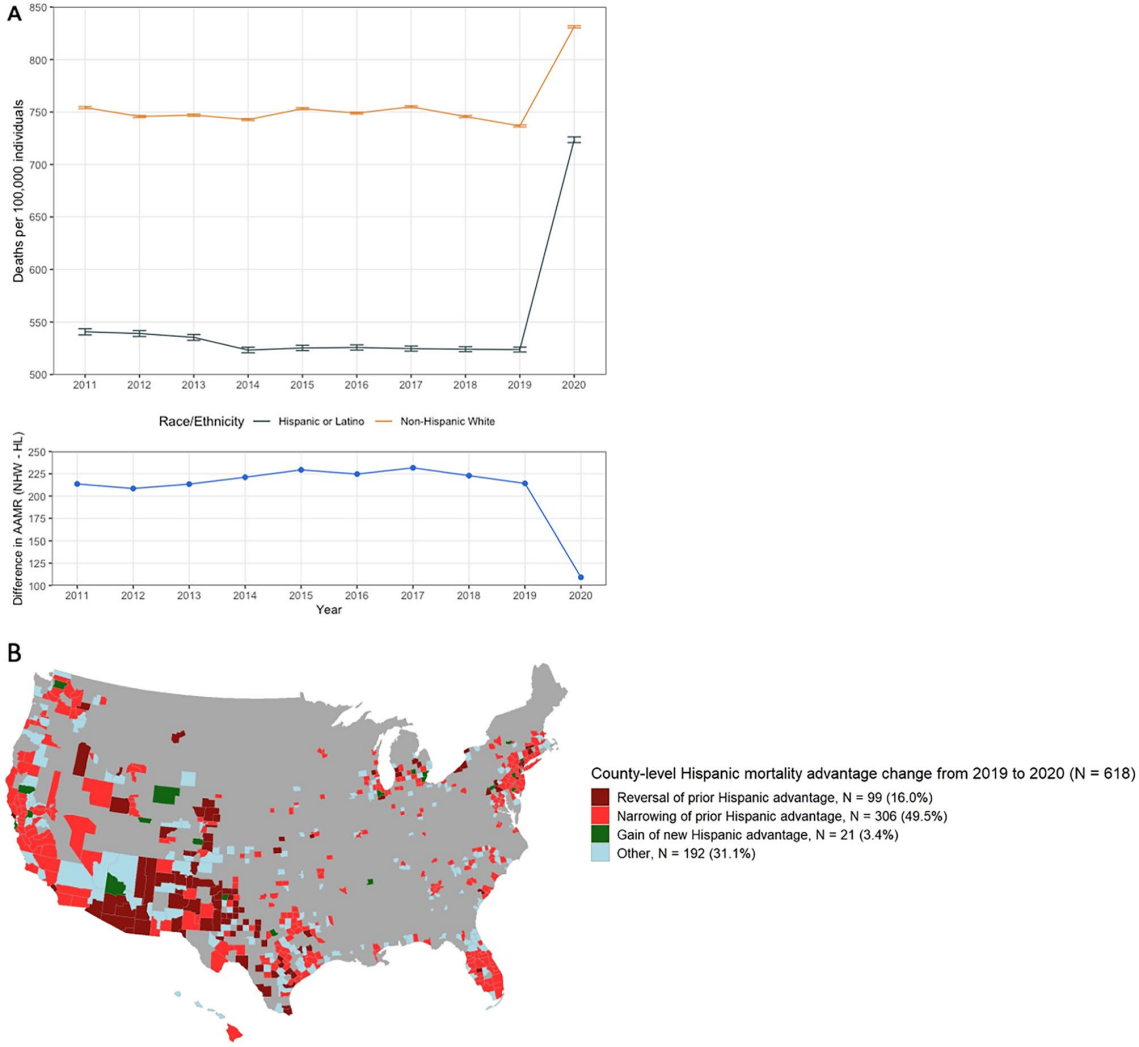
(**A**) National trends in Hispanic and NHW AAMRs in the US, 2011–2020. The first graph shows the individual Hispanic and NHW AAMRs (deaths per 100,000 individuals) and 95% confidence intervals by year. The second graph shows trends in the absolute national Hispanic mortality advantage (deaths per 100,000 individuals) by year when defined as the average NHW AAMR minus the average Hispanic AAMR. The previously steady national Hispanic mortality advantage, defined by the difference in the national NHW AAMR and Hispanic AAMR point estimates, decreased by approximately 50% from 2019 (214 fewer Hispanic vs NHW deaths per 100,000) to 2020 (109 fewer Hispanic vs NHW deaths per 100,000). (**B**) County-level trends in Hispanic vs NHW mortality in the US, 2019–2020. Of 618 counties, 99 (16%) experienced a reversal of a 2019 Hispanic mortality advantage in 2020 such that Hispanic mortality newly exceeded NHW mortality (average 2019 to 2020 change of 312 excess Hispanic vs NHW deaths per 100,000, 95% CI 274–348); 306 counties (50%) experienced a significant decrease in the magnitude of their 2019 Hispanic mortality advantage (average 2019 to 2020 change of 111 excess Hispanic vs NHW deaths per 100,000, 95% CI 99–124); and only 21 counties (3%) experienced a new Hispanic mortality advantage in 2020 such that NHW mortality was newly higher than Hispanic mortality (average 2019 to 2020 change of 290 fewer Hispanic vs NHW deaths per 100,000, 95% CI 235–345). The “other” counties group includes those with a 2019 Hispanic mortality advantage which further increased in 2020 and those with no Hispanic mortality advantage in both 2019 and 2020 (average 2019–2020 change of 67 fewer Hispanic vs NHW deaths per 100,000, 95% CI 48–87). Gray areas indicate lack of county-level data. This map was generated using RStudio, 1.4.1106 (http://www.rstudio.com/). Abbreviations: *AAMR* age-adjusted mortality rate, *NHW* Non-Hispanic White, *HL* Hispanic or Latino, *US* United States.

A previously steady Hispanic mortality advantage from 2011 to 2019 narrowed by approximately 50% from 2019 (214 fewer Hispanic vs NHW deaths per 100,000 individuals) to 2020 (109 fewer Hispanic vs NHW deaths per 100,000 individuals), due to a larger increase in deaths for Hispanic than NHW individuals (Fig. 1A). Hispanic individuals experienced higher COVID-19-specific mortality in 2020 versus NHW individuals (Table 1).

**Table 1:** Cause-specific Hispanic and Non-Hispanic White age-adjusted mortality in the US, 2019–2020.

2020 Leading Causes of Death	Hispanic					Non-hispanic white				
	2019		2020		2020–2019 AAMR ratio (95% CI)	2019		2020		2020–2019 AAMR ratio (95% CI)
	Deaths	AAMR (95% CI)	Deaths	AAMR (95% CI)		Deaths	AAMR (95% CI)	Deaths	AAMR (95% CI)	
COVID-19	0		65,237	155.5 (154.3, 156.7)		0		209,606	66.3 (66.0, 66.6)	
Heart disease	41,794	111.3 (110.2, 112.4)	48,323	122.7 (121.6, 123.9)	1.10 (0.34, 3.56)	513,673	165.8 (165.3, 166.2)	530,355	170.1 (169.6, 170.6)	1.03 (0.70, 1.52)
Malignant neoplasms	43,079	105.6 (104.6, 106.6)	43,942	103.6 (102.5, 104.6)	0.98 (0.37, 2.61)	462,064	151.4 (150.9, 151.8)	463,160	149.9 (149.4, 150.3)	0.99 (0.67, 1.47)
Unintentional injuries	18,874	35.1 (34.6, 35.6)	23,152	41.2 (40.7, 41.8)	1.17 (0.65, 2.11)	125,755	54.6 (54.3, 55.0)	141,335	62.8 (62.4, 63.1)	1.15 (0.78, 1.70)
Diabetes mellitus	10,166	25.6 (25.1, 26.1)	13,370	34.9 (34.3, 35.5)	1.21 (0.67, 2.18)	57,325	19 (18.9, 19.2)	64,098	21.1 (20.9, 21.3)	1.11 (0.91, 1.35)
Cerebrovascular diseases	11,959	32.8 (32.2, 33.4)	12,832	30.9 (30.4, 31.5)	1.06 (0.59, 1.91)	111,060	35.6 (35.4, 35.8)	116,752	37.1 (36.9, 37.3)	1.04 (0.86, 1.27)

Abbreviations: *AAMR* age-adjusted mortality rate, deaths per 100,000 individuals, *CI* confidence interval, *COVID-19* Coronavirus Disease 2019.

Across 618 counties, county-level Hispanic mortality increased significantly from 2019 to 2020 (average change of 73 excess Hispanic [vs NHW] deaths per 100,000, 95% CI 58–89). A total of 99 counties (16%) experienced reversal of their 2019 Hispanic mortality advantage such that Hispanic mortality began to exceed NHW mortality in 2020 (Table 2, Fig. 1B; average 2019–2020 change of 312 excess Hispanic deaths per 100,000, 95% CI 274–348). These counties had a high average county-level Hispanic representation of 36% (95% CI 32–41) in 2020. An additional 306 counties (50%) experienced a post-pandemic decrease of their 2019 Hispanic mortality advantage due to excess Hispanic death rates (average 2019–2020 change of 111 excess Hispanic vs NHW deaths per 100,000, 95% CI 99–124). These 306 counties had an average Hispanic representation of 22% (95% CI 15–29).

**Table 2:** US county-level changes in Hispanic versus Non-Hispanic White age-adjusted mortality categorized according to effect on the Hispanic mortality advantage, 2019–2020.

Category	N (%) counties (total = 618)	Average county-level 2019–2020 change (95% CI)
2020 reversal of prior 2019 Hispanic mortality advantage	99 (16)	312 excess Hispanic vs. NHW deaths per 100,000 (274–348)
2020 narrowing of prior 2019 Hispanic mortality advantage	306 (50)	111 excess Hispanic vs. NHW deaths per 100,000 (99–124)
2020 gain of new Hispanic mortality advantage	21 (3)	290 fewer Hispanic vs. NHW deaths per 100,000 (235–345)
Other*	192 (31)	67 fewer Hispanic vs NHW deaths per 100,000 (48–87)

Abbreviations: *NHW* Non-Hispanic White.

*Other—includes those with a 2019 Hispanic mortality advantage which further increased in 2020 and those with no Hispanic mortality advantage in both 2019 and 2020.

Only 21 counties (3%) had a new Hispanic mortality advantage in 2020 wherein Hispanic mortality fell below NHW mortality (average 2019–2020 change of 290 fewer Hispanic vs NHW deaths per 100,000, 95% CI 235 – 345). These 21 counties had an average Hispanic representation of 17% (95% CI 12–23). In the remaining 192 “other” counties (31%) with available data, there was an average county-level Hispanic representation of 20% (95% CI 13–28), with an average 2019–2020 change of 67 fewer Hispanic vs NHW deaths per 100,000 (95% CI 48–87).

## Discussion

In 2020, there was substantial reduction of a previously steady Hispanic vs NHW mortality advantage in the US, likely driven by greater Hispanic COVID-19-related mortality. Two-thirds of analyzed US counties experienced reversal or decrease of a pre-pandemic Hispanic mortality advantage. The disproportionate burden of the COVID-19 pandemic on Hispanic populations may have thus newly altered the well-established Hispanic paradox and may portend a US-wide Hispanic health crisis beyond 2020 if the underlying drivers remain unchecked^8^.

Future studies should urgently evaluate the individual and county-level determinants of these concerning race/ethnicity-specific trends. For example, counties that experienced reversal of a Hispanic mortality advantage in 2020 had the greatest increase in excess Hispanic vs NHW death rates from 2019 to 2020—and had higher Hispanic representation compared with other counties and compared with national US Census representation^9^. This observation is consistent with prior associations between greater county-Hispanic density and elevated mortality^10^. Disentangling these associations in the context of relevant socioeconomic and health variables may help better understand the actionable individual and county-level determinants of the concerning trends observed in the present study. These analyses should be pursued in dedicated follow-up studies of reliable databases that report relevant demographic, socioeconomic, health, and cultural variables. Disaggregated Hispanic subgroup data may further unmask important heterogeneity^11^.

Our study supports the disproportionate burden of COVID-19 among Hispanic populations, nationally. These findings should be interpreted in the context of ethnicity-specific disparities stemming from the COVID-19 pandemic, including higher COVID-19 positivity, hospitalizations, and deaths, along with links between COVID-19 and the disease conditions that form the most common causes of death in the US. For example, in a retrospective analysis of 7,868 patients hospitalized with COVID-19, Hispanic patients experienced a higher burden of morbidity and mortality due to disproportionately higher rates of COVID-19 hospitalizations^6^. A systematic review showed disproportionately higher COVID-19 related mortality in Hispanic populations^12^. In a large health system in Northern California, Hispanic populations were more likely than non-Hispanic populations to test positive for SARS-CoV-2^13^. A recent study of US-wide age- and sex-adjusted deaths further found that Hispanic populations experienced a rise in heart disease and cerebrovascular disease deaths in March-August 2020 as compared with March–August 2019^14^. In a study of cancer patients, Hispanic individuals experienced significantly higher risk for COVID-19 infections as compared with NHW individuals^15^.

Potential explanations for these findings include Hispanic overrepresentation in frontline or essential roles; a higher likelihood of living in denser communities and multigenerational households, making it more challenging to isolate and contain exposure risk; differential burden of underlying comorbidities such as diabetes; and language differences that may limit access to public health messaging^13^. The links between COVID-19 and downstream effects on the traditional leading causes of death in the US may lead to long-term disparities beyond the pandemic if their structural underpinnings remain unaddressed. In addition, future COVID-19 endemicity in the US may continue to drive long-term COVID-19 ethnic disparities in disease burden and mortality. The role of structural racism in propagating these disparities should be closely examined and mitigating efforts should be strengthened^16^.

Study limitations include lack of county-level data in several counties due to small Hispanic populations and potential misclassification or misreporting of mortality and race/ethnicity data during the pandemic.

## Materials and methods

Using the Center for Disease Control Wide-ranging Online Data for Epidemiologic Research (CDC WONDER) tool, we obtained US national and county-level estimates for Hispanic/Latino (Hispanic) and NHW age-adjusted mortality rates (AAMRs, deaths per 100,000 individuals) and corresponding standard errors from 2011 to 2020. For county-level analyses, counties were included if both Hispanic and NHW AAMRs were available and yearly Hispanic and NHW deaths were 20 or greater. The National Center for Health Statistics uses the year 2000 US population and the direct method to calculate AAMRs.

We estimated national and county-level excess race/ethnicity-specific mortality in 2019 and 2020 as the absolute difference between NHW and Hispanic AAMRs (delta-AAMR). A Hispanic mortality advantage was defined as the NHW AAMR exceeding the Hispanic AAMR. We calculated absolute differences between 2020 and 2019 delta-AAMRs. Based on the 2019 to 2020 change in the delta-AAMR, we categorized counties into 4 groups: (1) those that experienced a 2020 reversal of a 2019 Hispanic mortality advantage such that Hispanic mortality newly exceeded NHW mortality in 2020, (2) those that experienced a 2020 decrease of a 2019 Hispanic mortality advantage, (3) those that experienced a new 2020 Hispanic mortality advantage such that NHW mortality newly exceeded Hispanic mortality in 2020, and (4) an “other” group that included all remaining counties with available data, including those that experienced a 2020 increase in a 2019 Hispanic mortality advantage and those with no Hispanic mortality advantage in both 2019 and 2020. Within each group, we calculated the average county-level change in delta-AAMRs (indicating changes in relative Hispanic vs NHW mortality rates) from 2019 to 2020 and estimated 95% confidence intervals (CIs) using standard methods with the mean, standard deviation, sample size, and a standard coefficient (defined as 1.96). Similarly, we calculated the average county-level Hispanic representation with 95% confidence intervals in these groups.

We obtained national cause-specific Hispanic and NHW AAMRs in 2019 and 2020 for COVID-19 and the 5 leading causes of death. We calculated 2020 to 2019 AAMR risk ratios and 95% CIs^5^. Results were considered statistically significant for differences if the 95% CIs did not cross zero and for comparisons if 95% CIs did not overlap. Analyses were performed using RStudio, 1.4.1106. Geospatial mapping was performed with Federal Information Processing System (FIPS) codes using RStudio, 1.4.1106.

## Data Availability

The national and county-level age-adjusted mortality data that support the findings of this study are available from Center for Disease Control Wide-ranging Online Data for Epidemiologic Research system (CDC WONDER), https://wonder.cdc.gov/ucd-icd10.html.
